# The Action Constraints of an Object Increase Distance Estimation in Extrapersonal Space

**DOI:** 10.3389/fpsyg.2019.00472

**Published:** 2019-03-06

**Authors:** Romàn Viçenç Josa, Thomas Camus, Vincent Murday, Nicolas Morgado, Richard Palluel-Germain, Lionel Brunel, Denis Brouillet

**Affiliations:** ^1^Department of Psychology, Epsylon, Paul Valéry University of Montpellier, Montpellier, France; ^2^Department of Psychology, Julius-Maximilians-Universität Würzburg, Würzburg, Germany; ^3^Centre de Recherches sur le Sport et le Mouvement, University of Paris Nanterre, Nanterre, France; ^4^LPNC, Université Grenoble-Alpes, Grenoble, France; ^5^LPNC, CNRS, Grenoble, France

**Keywords:** distance estimation, action constraints, extrapersonal space, allocentric reference frame, spatial perception

## Abstract

This study investigated the role of action constraints related to an object as regards allocentric distance estimation in extrapersonal space. In two experiments conducted in both real and virtual environments, participants intending to push a trolley had to estimate its distance from a target situated in front of them. The trolley was either empty (i.e., light) or loaded with books (i.e., heavy). The results showed that the estimated distances were larger for the heavy trolley than for the light one, and that the actual distance between the participants and the trolley moderated this effect. This data suggests that the potential mobility of an object used as a reference affects distance estimation in extrapersonal space. According to embodied perception theories, our results show that people perceive space in terms of constraints related to their potential actions.

## Introduction

According to various theoretical approaches, visual space perception depends in part on action constraints [i.e., the phenotypic account (Proffitt and Linkenauger, 2013); the action-specific account (Witt and Riley, 2014); and the evolved navigation theory (Jackson and Willey, 2011)]. Sparrow and Newell (1998) refer to action constraints as every property of an organism (e.g., morphology, physiology, and behavior), a task (e.g., explicit rules, tool properties, and biomechanical rules), and/or the environment (e.g., obstacles and topographical variations) defining the action potentialities of an organism. Despite some disparities, the action constraint theories (ACT) of perception all claim that visual space perception is embodied (Coello and Delevoye-Turrell, 2007; Proffitt, 2013), meaning that body-based information plays a major role in perceptual processes. For a more detailed presentation of these theories, see Morgado and Palluel-Germain (2015).

This approach is debated, however, and alternative theories of spatial perception consider the influence of action constraints to be primarily effective at the response stage rather than at the perceptual stage (Hutchison and Loomis, 2006; Durgin et al., 2009, 2011, 2012; Firestone and Scholl, 2016; for a review, see Philbeck and Witt, 2015). Nevertheless, King et al. (2017) have recently presented new empirical evidence, as well as strong theoretical arguments, for the claim that action constraints have a genuine effect on space perception (see also Philbeck and Witt, 2015). The aim of the present study was not to wind up this debate but to investigate the effects of action constraints on allocentric distance estimation in extrapersonal space. More precisely, we were interested in the effect of an object used as an allocentric reference frame on distance estimation. Throughout this article, we refer to an allocentric reference frame, following Fini et al. (2015), as an object used as a reference for estimating a distance between two objects which are independent of the perceiver’s body.

The literature has shown that space perception can be altered by variations in behavioral capabilities (Witt and Sugovic, 2010; Taylor et al., 2011), physiological state (Schnall et al., 2010; Proffitt and Linkenauger, 2013; White et al., 2013), tool-use (Kirsch et al., 2012; Osiurak et al., 2012; Morgado et al., 2013; Bourgeois et al., 2014), or social support (Fini et al., 2015, 2017). For example, someone’s peripersonal space can be increased when using a tool that enlarges one’s reaching capabilities (Farnè and Làdavas, 2000; Maravita et al., 2001; Serino et al., 2007; Costantini et al., 2011b). The mere presence of others can also enlarge one’s reaching capabilities (Costantini et al., 2011c; Cardellicchio et al., 2013). Recently, similar effects have also been found for extrapersonal space (Fini et al., 2014, 2015, 2017). In a 3D virtual environment, Fini et al. (2015) asked participants to estimate the location (“Near” or “Far”) of a target object located at progressively increasing or decreasing distances from an instructed reference frame. The reference frame was either a virtual human agent or a static object. They found that participants estimated that the target was closer to the agent than to the static object. More interestingly, the results showed that this effect was observable only when the virtual human body was free to move, but not when it was tied to a pole with a rope. These results suggest that using a virtual agent (with movement capabilities) as a reference frame for space categorisation triggers a representation of the action potentialities offered by the environment. Fini et al. (2015) therefore shed a new light not only on the effect of action constraints on distance in extrapersonal space, but also on the effect of the reference frame. This conclusion is in line with several studies suggesting that people tend to automatically adopt other people’s visual perspective when making judgments about their direct environment (Tversky and Hard, 2009; Samson et al., 2010; Surtees and Apperly, 2012). As Fini et al. focused on the comparison between a virtual agent and static objects, however, it is not yet known whether this spatial remapping holds when the allocentric reference frame is a non-human object with action potentialities.

The objective of the present study was to fill this gap in the literature by manipulating the action constraints of a mobile object used as an allocentric reference frame. We hypothesized that when people intend to push an object toward another object located in their extrapersonal space, they perceive the distance between these two objects depending on the anticipated effort needed to move the first object. To test this hypothesis, we designed two experiments in which participants had to estimate several distances between a library trolley and a target, both being in the participant’s extrapersonal space. The library trolley served as an allocentric reference frame. We manipulated the trolley weight by having an empty trolley (i.e., light trolley) and a loaded trolley (i.e., heavy). We manipulated this variable between-subject in Experiment 1 and within-subject in Experiment 2. Experiment 1 took place in a real environment (i.e., a corridor in a library), whereas Experiment 2 took place in a virtual 3D scene (i.e., images representing similar scenes as in Experiment 1). Due to action constraints related to the trolley weight, we expected that the participants would estimate the distances between the trolley and the target as further when the trolley was heavy, than when it was light.

## Experiment 1

In order to reduce the bias related to potential demand characteristics (Durgin et al., 2009), we manipulated the trolley weight in a between-subject design. The objective of this manipulation was to avoid that participants would be compliant with the experimental task demands. In this case, each participant experienced only one condition (i.e., one level of action constraint) and therefore should not be able to somehow strategically adjust her performance according to another condition.

### Methods

#### Participants

Forty students from the University Paul Valery of Montpellier, France (21 females) participated (*m*_age_ = 23.5, *SD*_age_ = 3.06). All participants read and signed a written informed consent about the experimental protocol, which was approved by the local ethics committee. All participants had normal or corrected-to-normal vision as indicated by self-report. They were *a priori* naïve to the purpose of the experiment and they did not participate in prior distance-perception experiments.

#### Apparatus and Procedure

**FIGURE 1:**
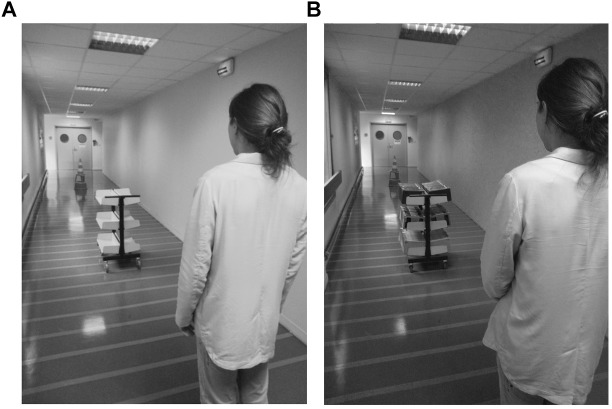
Experimental apparatus of Experiment 1 from the participants’ perspective for the light group **(A)** and the heavy group **(B)**. These pictures represent the near P-T distance condition, with the trolley located 3 m from the participant and the cone (target) located 8 m away from the trolley. Written and informed consent about the potential publication of these images was obtained from the individual appearing on the figure.

The experiment took place in a 15-m-long and 2.5-m-wide corridor. The participants were randomly assigned to the light-trolley group or to the heavy-trolley group. In the light-trolley group, the trolley was empty and weighted 12 kg. In the heavy-trolley group, the trolley was filled with books and weighted nearly 170 kg (see Figure 1). The participants had to estimate allocentric distances between the trolley and a cone (i.e., T-C distances) aligned with their midsagittal axis in two conditions depending on the trolley distance to the participants (i.e., P-T distances). In the near condition, the trolley was at 3 or 4 m from the participants, and the T-C distances that the participants had to estimate were equal to 5, 6, 7, and 8 m. In the far condition, the P-T distance was equal to 6 or 7 m, and the T-C distances that the participants had to estimate were equal to 3, 4, 5, and 6 m. We varied the distances to prevent the participants to anchor their estimations in one condition on their estimations in another condition. Both the P-T distances and T-C distances varied randomly within-subject from one trial to another. The participants completed a total of 12 trials, including four practice trials and eight test trials (one test trial^∗^four T-C distances^∗^two trolley’s positions). For these practice trials, the P-T distance could be equal to 3, 4, 5, or 6 m and the T-C distance could be equal to 4, 5, 7, or 8 m. At the beginning of each trial, the participants turned back while the experimenter set the trolley and the cone at a selected distance by using small marks on the floor. We empirically determined the size of these marks so that they were unnoticeable from the participants’ position. The participants then turned back again to face the trolley and verbally estimated the T-C distance in meters without time limit. The participants had to stand at the same location throughout the experiment without leaning to one side.

Before starting the experiment, the experimenter indicated to the participants that they would have to estimate all the T-C distances as spontaneously as possible, to cover all the T-C distances while walking and pushing the trolley, and to estimate all the T-C distances again. We gave this instruction to lead participants to anticipate the effort needed to push the trolley (Witt et al., 2004). However, at the end of the test, the participants did not push the trolley and did not estimate the T-C distances again. Finally, the experimenter recorded the participants’ height with a tape measure at the end of the experiment (*m*_heavy-group_ = 172.6 cm, *SD* = 8.96; *m*_light-group_ = 170.2, *SD* = 7.69).

### Results

We computed the median estimated distance per condition for each participant (regarding the use of similar method, see Kirsch et al., 2017). Moreover, given that distances for the near position and for the far position were different, we computed a bias ratio expressing the medians of the estimated distances as a ratio of medians of the actual distances to compare estimations in near and far P-T distances (see the Supplementary Tables S1, S2, available online). A bias ratio of 1 means that the participants estimated the distances perfectly. A bias ratio above 1 or below 1 means that the participants overestimated or underestimated the distances, respectively. We discarded from our analysis the participants who showed inconsistent mean bias ratio between near and far P-T distances as indicated by a difference between these conditions equal to or larger than plus-or-minus 3 *SD*. This led us to exclude one participant in each group.

**FIGURE 2:**
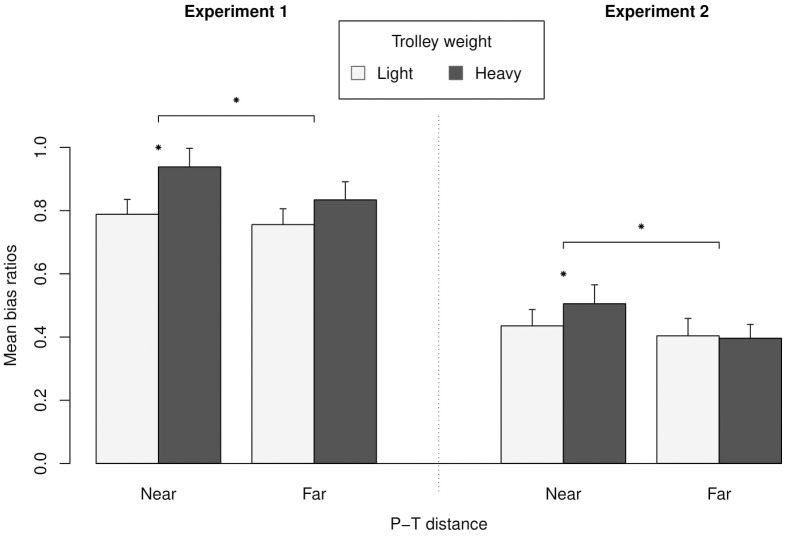
Mean bias ratio expressing the median estimations as a ratio of the actual median distances depending on trolley weight and the P-T distance factors. A bias ratio above 1 or below 1 means that the participants overestimated or underestimated the distances, respectively. Error bars indicate one SEM. ^∗^*p* ≤ 0.05.

We ran a 2 × 2 mixed-designed analysis of variance (ANOVA) with the trolley weight as a between-subject independent variable and P-T distance as a within-subject independent variable. The dependent variable was the bias ratio. This analysis revealed a significant Trolley Weight × P-T Distance interaction, *F*(1,36) = 4.2, *p* = 0.047, $\eta_{p}^{2}$ = 0.10 (Figure 2), and a significant main effect for the P-T distance, *F*(1,36) = 15.3, *p* < 0.001, $\eta_{p}^{2}$ = 0.298. The main effect of weight was not significant, *F*(1,36) = 2.4, *p* = 0.13, $\eta_{p}^{2}$ = 0.062.

An *a priori* contrast analysis showed that, when the P-T distance was near, participants from heavy-trolley group (*m*_near/heavy_ = 0.94, *SD*_near/heavy_ = 0.26, *N* = 19) estimated that the T-C distance was larger than participants from light group (*m*_near/light_ = 0.79, *SD*_near/heavy_ = 0.21, *N* = 19), *F*(1,36) = 3.95, *p* = 0.05, $\eta_{p}^{2}$ = 0.10. According to our data, the more plausible value for this effect in the population was *m*_heavy trolley-lighttrolley_ = 0.15, 95% CI for μ_heavy trolley-light trolley_ [0.00, 0,30]. The contrast analysis also showed that this difference vanished when the P-T distance was far, participants from heavy group (*m*_far/heavy_ = 0.83, *SD*_far/heavy_ = 0.25, *N* = 19) did not statistically estimate larger distances than participants from the light group (*m*_far/light_ = 0.76, *SD*_far/light_ = 0.22, *N* = 19), *F*(1,36) = 1.05, *p* = 0.31, $\eta_{p}^{2}$ = 0.03, which accounts, in part, for the absence of a significant main effect of weight. Finally, no correlation was found between the estimations and the height of the participants (*r* = 0.06).

### Discussion Experiment 1

For a near P-T distance, participants estimated that the T-C distance was significantly longer when the trolley was heavy than when it was light. For far P-T distance, this effect decreased and was not statistically significant. These results suggest that the trolley weight effect on distance estimation depends on the P-T distance. This interpretation is consistent with the action-specific account of perception, according to which action potentialities affect space perceptions.

Alternative explanations of our results cannot be ruled out. For instance, one could argue that this effect might arise from the fact that participants anticipated covering a longer average distance while pushing the trolley in the near condition than in the far condition. Indeed, as these conditions differed in terms of actual T-C distances, the participants anticipated pushing the trolley for 65% of the total average distance that they had to cover in the near condition and only for 41% of the total average distance that they had to cover in the far condition. Thus, pushing the trolley required more effort in the near condition than in the far condition, which could explain our results.

Another explanation of the interaction effect could be that visual variables, rather than action constraints, are the sources of the observed differences between the light- and heavy-trolley groups. Indeed, the floor was more occluded by the heavy trolley, which was full of books and with its top being higher in the visual field, than by the light one that was empty. The visibility of the ground plane and the angular declination of the gaze are both known to play a role in distance perception (Ooi et al., 2001), however, if it was the case, we should have observed a larger difference between the heavy and the light trolley groups when the P-T distance was far than when it was near, because the heavy trolley occluded a larger part of the T-C distance. It also seems somewhat counterintuitive to overestimate a partially occluded distance because it would have meant that they overcompensated to account for the occluded portion of the T-C distance. Indeed, experimental arguments have been provided by He et al. (2004) showing that when the ground surface between an observer and a target is disrupted by an occluding object, this leads to egocentric distance underestimation. We think, therefore, that we can rule out this visual interpretation.

## Experiment 2

The results of Experiment 1 revealed a statistically significant Trolley Weight × P-T distance interaction. Given that the effect observed was rather small, some reservations remain whether it really reflected the influence of the manipulated factors. Also, and as claimed earlier, it is possible that participants have anticipated covering a longer average distance while pushing the trolley in the near P-T distance than in the far P-T distance. This confound could compromise the internal validity of our conclusions. Given these limitations, Experiment 2 was a conceptual replication of Experiment 1. Moreover, as people generally make larger distance underestimations in virtual environments than in real environment (Creem-Regehr et al., 2005; Armbrüster et al., 2008), we aimed to extend the conclusions about our effect of interest to virtual environments. Thus, using virtual images instead of real distances and objects allowed us to (1) keep the visual inputs constant across participants, (2) use the same T-C distances in near and far P-T distances, and (3) increase the number of estimations for each distance. Finally, because the lack of power in Experiment 1 is partially due to our between-subject manipulation of the trolley weight, we used a within-subject design in Experiment 2, with systematic order effect addressed by randomization.

### Methods

#### Participants

Given the effect size reported in Experiment 1 ($\eta_{p}^{2}$ = 0.10), the required sample size for Experiment 2 was determine by conducting an *a priori* power analysis using G^∗^Power software (version 3.1; Faul et al., 2009). The analysis indicated that a minimum sample size of 14 participants was required in this study to detect a medium to large effect size with an adequate power (1 – ß > 0.80) and an alpha of 0.05. Following this, fifteen students from the University Paul Valery of Montpellier, France, participated (*m*_age_ = 21.7, *s*_age_ = 3.8, nine females and six males). All participants had normal or corrected-to-normal vision as indicated by self-report. They were *a priori* naïve to the purpose of the experiment and they did not participate in prior distance-perception experiments. All participants read and signed a written informed consent about the experimental protocol, which was approved by the local ethics committee.

#### Apparatus and Procedure

**FIGURE 3:**
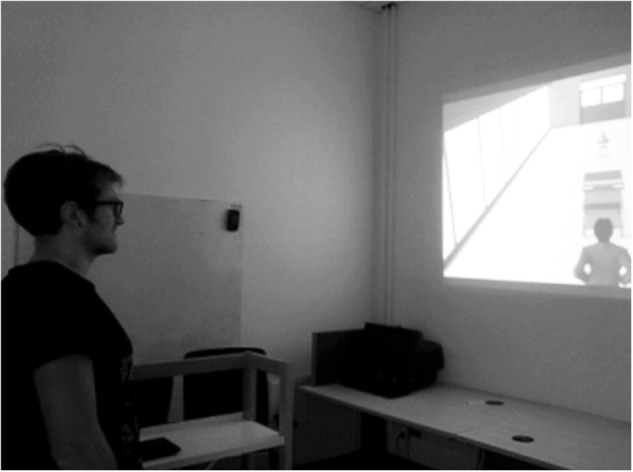
Experimental apparatus of Experiment 2 from the participants’ perspective for the light condition. This picture represents the near P-T distance condition, with the light trolley located 3 m from the participant. Written and informed consent about the potential publication of these images was obtained from the individual appearing on the figure.

**FIGURE 4:**
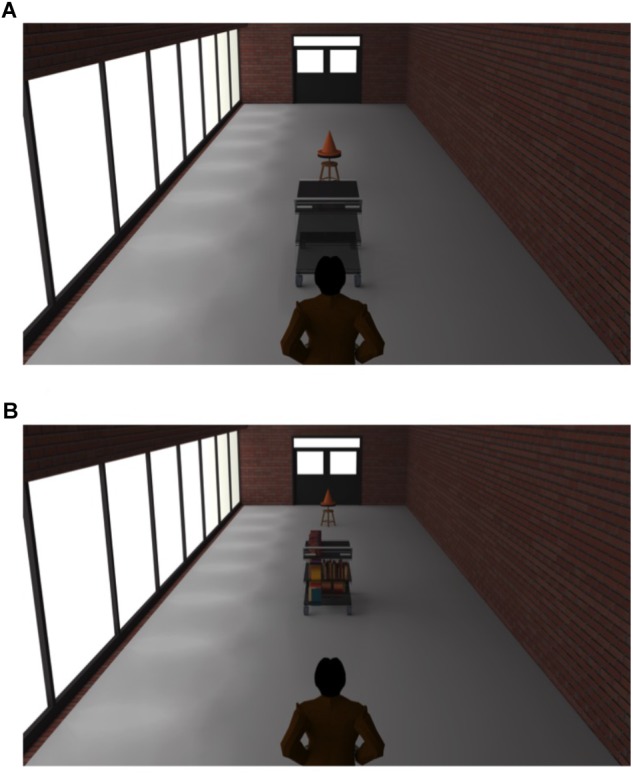
3D images used as stimuli in Experiment 2. Image **A** represents the near P-T distance in light condition, with the trolley located 3 m from the avatar and the cone located 3 m away from the trolley. Image **B** represents the far P-T distance in the heavy condition, with the trolley located 6 m from the avatar and the cone located 7 m away from the trolley.

The experiment took place in an experimental room (3.15-m-long and 3-m-wide). A video projector (Epson EB-U04 Tri-LCD) projected the 20 images of a virtual 3D environment depicting allocentric T-C distances on a wall located at 2.5 m from the participants (Figure 3). The images were designed with Archicad 18 and Artlantis 6. The size of the projected images was 108 cm × 180 cm. Each image represented a third person scene were an avatar was standing in a corridor with a trolley and a cone aligned with his midsagittal axis (Figure 4). We chose a third-person view because it appears there is no apparent gain of immersion from first- over third-person view in video games (Black, 2017). On half of the images the trolley was empty (i.e., light trolley) and on the other half the trolley was full of books (i.e., heavy trolley). The trolley was at 3 and 6 m from the avatar in the near and far P-T distance, respectively. The T-C distances varied from 3 to 7 m (five distances with a step of 1 m). The participants had to estimate four times each T-C distance in each experimental condition (2 Trolley Weights × 2 P-T Distances × 5 T-C distances × 4 Blocks × 1 Trial). Within each block, the T-C distance, the P-T distance and the trolley weight randomly varied within subject from one trial to another. For each trial, the participants had to verbally estimate T-C distance with no time limit. Then, they had to press the space bar on a keyboard positioned on their left side to start the next trial. We used the same cover story as in Experiment 1 by telling the participants that they would have to actually push the trolley afterward.

### Results

We used the same statistical procedure as in Experiment 1 to compute our bias ratio and to discard inconsistent data. This led to the exclusion of two participants. We also ran a 2 × 2 within-subject ANOVA with trolley weight and P-T distance as within-subject independent variables. The dependent variable was the bias ratio for each condition. This analysis revealed a statistically significant Trolley Weight × P-T Distance interaction, *F*(1,12) = 5.1, *p* = 0.04, $\eta_{p}^{2}$ = 0.30 (Figure 2), a statistically significant main effect of trolley weight, *F*(1,12) = 7.3, *p* = 0.02, $\eta_{p}^{2}$ = 0.38, and statistically main effect of P-T distance, *F*(1,12) = 6.4, *p* = 0.03, $\eta_{p}^{2}$ = 0.35. An *a priori* contrast analysis showed that, when the trolley was near, participants in the heavy-trolley condition (*m*_near/heavy_ = 0.51, *SD*_near/heavy_ = 0.22, *N* = 13) estimated that the T-C distance was larger than participants in the in the light-trolley condition (*m*_near/light_ = 0.44, *SD*_near/heavy_ = 0.19), *F*(1,12) = 11.6, *p* = 0.005, $\eta_{p}^{2}$ = 0.49. According to our data, the more plausible value for this effect in the population was *m*_heavy trolley-light trolley_ = 0.07, 95% CI for μ_heavy trolley-light trolley_ [0.03, 0,11]. The contrast analysis also showed that this difference vanished when the P-T distance was far as participants did not estimated longer distances in the heavy-trolley condition (*m*_far/heavytrolley_ = 0.40, SD_far/heavy_ = 0.16) than in the light-trolley condition (*m*_far/lighttrolley_ = 0.40, *SD*_far/light_ = 0.20), *F*(1,12) = 0.14, *p* = 0.72, $\eta_{p}^{2}$ = 0.00 (see the Supplementary Tables S1, S2, available online).

### Discussion Experiment 2

One of the goals of this second experiment was to replicate with virtual stimuli what was found in Experiment 1. For a near P-T distance, participants estimated that the T-C distance was significantly longer when the trolley was heavy than when it was light. For far P-T distance, this effect disappeared and was not statistically significant. Despite this Trolley Weight × P-T Distance interaction, the results of this experiment differ from those of Experiment 1 for two reasons. First, the main effect of trolley weight was statistically significant in Experiment 2, even though it was not in Experiment 1. Given that the unstandardised trolley weight effect size was stronger in Experiment 1 (*m*_heavy trolley-light trolley_ = 0.09) than in Experiment 2 (*m*_heavy trolley-light trolley_ = 0.03), this difference in statistical significance is likely due to the unaccounted between-subject variability in the between-subject design. Second, participants underestimated the distances to a larger degree than in Experiment 1 (less than half the actual distances). This was consistent with previous studies showing that distance perception is more compressed in a virtual environment than in real life (Thompson et al., 2004; Creem-Regehr et al., 2005). More importantly, our results indicate that despite a larger bias in distance estimation, the pattern of results from Experiment 2 was consistent with those of Experiment 1. This suggests that our conclusions might hold for the real environment as well as virtual ones. We believe that such similarities in results could be explained by the reliable sense of presence provided by the third-person perspective (Draper et al., 1996; Thompson et al., 2004; Creem-Regehr et al., 2005), but this interpretation would need further investigation.

## General Discussion

As shown in numerous studies, action performance (Witt and Dorsch, 2009) or social factors can bias the estimation of allocentric extent within extrapersonal space (Fini et al., 2014, 2015). In the present study, we investigated the role of action constraints related to an object used as a reference on the estimation of allocentric distances. For this purpose, we designed an experiment in which participants estimated the distances between a trolley and a cone (Experiment 1) while believing that they would push the trolley later. The results showed that the participants estimated longer distances when the trolley was heavy (i.e., loaded with books) than when it was light (i.e., empty). Importantly, such an impact was moderated by the location of the trolley with regard to the participants. Finally, we observed similar results with virtual stimuli (Experiment 2).

Our interpretation of this result is that the anticipated effort required to push the trolley affected the way participants perceived the distance between the trolley and the cone. This interpretation is consistent with the ACT, which claims that action constraints affect visual perception of space (Proffitt and Linkenauger, 2013; Witt and Riley, 2014; Morgado and Palluel-Germain, 2015; Zadra et al., 2016). Our analysis also revealed that the trolley weight affected distance perception only when the trolley was near the participants. This suggests that we met a boundary condition of the effect of the allocentric reference frame – and its related action constraints – on space perception.

### The Role of the Reference Frame Characteristics

Some studies have shown that people spontaneously adopt other people’s perspectives when judging space (Tversky and Hard, 2009; Samson et al., 2010; Surtees and Apperly, 2012). For instance, people might take into account the potential movements of others to judge whether a target located in extrapersonal space is near or far from themselves (Fini et al., 2015). One reason for adopting another person’s perspective is the common mapping of one’s own and the other’s motor potentialities (Tversky and Hard, 2009; Samson et al., 2010; Surtees and Apperly, 2012), which can be explained by the remapping of one’s space representation depending on the potential actions of others. As mentioned by Fini et al. (2015), however, it is possible that the human body could affect space perception as a tool with motion opportunities and not necessarily because it is a human reference frame. To answer this question, we used a non-human object with motion potentialities as a reference frame and we tested whether people could remap their space perception according to these potentialities. Our results indicated that distance estimations were indeed different depending on the reference frame characteristics (i.e., trolley weight). Considering that this characteristic has a direct impact on the way someone might plan to interact with an object, it seems likely that they will also integrate it as physical constraint in their own motor potentialities. As tool-use affects perceived distances (Witt et al., 2005), extrapersonal space could also be processed according to the potential actions offered by an allocentric reference frame, which would contribute to scaling the environment to the bioenergetic resources required to traverse the distances (Zadra et al., 2016).

The results of these experiments also revealed that trolley weight affected the participants’ estimations only when the trolley was near them. This suggests that the participants did not integrate the physical constraints of the allocentric reference frame for the far P-T distance. This interpretation is consistent with studies showing that motor simulation and affordances are spatially constrained (Costantini et al., 2010, 2011a), which implies that, depending on the reachability of an object, their perception activates different neural processes, in particular certain motor processes (Rizzolatti et al., 1996; Gallese, 2016). Thus, depending on their spatial relationship with an object, people would not use the same neural patterns when planning to interact with it. We therefore propose that an allocentric reference frame with motion opportunities would lead to different distance estimations of the extrapersonal space depending on such factors as (1) the physical effort needed to move it, and (2) its location in reference to the viewer. We cannot, however, exclude the possibility that the interaction effect is due to alternative explanations and this first interpretation would benefit from further experimental replications.

### Alternative Explanations

The experience of perception is known to resist researchers’ attempts to directly measure it in behavioral and neuropsychological studies. An important theoretical and experimental debate is still ongoing regarding whether action genuinely affects either perceptual or post-perceptual processes (for a review, see Philbeck and Witt, 2015). Among the different questions raised by this debate, the one that we are interested in here is whether higher-level cognitive and/or bodily states can “penetrate” perception (Firestone and Scholl, 2016). In other words, whether what one sees is a combination of both bottom-up factors and one’s beliefs, linguistic representations, or action performances.

This question could be asked regarding our results, because the participants performed verbal estimations, which can be affected by both perceptual differences and response-based processes (Poulton, 1979; Witt et al., 2016). For example, one could argue that if participants truly engaged in a motor simulation process and simulated walking with the trolley before their estimations, it would not necessarily mean that perception itself (i.e., visual processing) was altered. The participants could have transposed the number of steps needed to cover the different distances and based their estimations on this mental simulation. Also, and apart from the perceived effort of pushing, this mental process could have led participants to bias their judgments depending on temporal estimates (i.e., the estimated time to move the trolley to the cone).

Visual perception is known to rely on various sources of information, including visual information (Cutting and Vishton, 1995), physiological information (White et al., 2013; Witt and Riley, 2014), action intentions (Witt et al., 2010, 2004, 2005), as well as on multisensory integration processes (Campos et al., 2012, 2014; Kirsch et al., 2017). More precisely, these works show that visual and bodily variables are differently weighted during the estimation of space or object size, depending on the available sources of information. Both the intention to push the trolley and the anticipation of the effort therefore seem likely to be involved in the perceptual process. The extent to which this bodily variable can be accounted for in the final estimation remains, however, an open question.

Witt et al. (2018): see also (Witt and Sugovic, 2013; King et al., 2017; Witt, 2017) recently provided strong experimental arguments in favor of the action-specific approach of perception. Using the Pong task experiment, they showed that when participants were explicitly told the hypothesis and instructed to resist the effect of their ability to block the ball, their ability still affected their perception of the ball’s speed. Those results highlight that visual experience seems affected by one’s ability to act, as well as by the consequences of one’s actions in the environment. More importantly, such findings not only refute the idea of reducing visual experience to mere visual processes, but also question the relevance of the perceptual/post-perceptual distinction when studying the experience of perceiving.

## Conclusion

We observed that a heavy trolley used as an allocentric reference frame led participants to estimate longer distances than a light trolley. This distinction was only observed when the trolley was located near the participants and not when it was far from them. This therefore suggests that during visual space perception, an allocentric reference frame with motion potentialities can constrain distance estimation in extrapersonal space. Such results are in line with previous studies showing the effects of action constraints on distance perception (Stefanucci and Geuss, 2009; Witt, 2011; Proffitt and Linkenauger, 2013; Morgado et al., 2013; King et al., 2017) and suggest going further by considering an external and a non-living reference frame as a potential “tool” that could increase or decrease people’s action opportunities. The moderation effect of the P-T distance also suggests that the integration of the potentialities offered by an allocentric reference frame is space-dependent. These findings are consistent with an embodied view of perception (Proffitt, 2013) and contribute to emphasizing the relevance of taking into account both visual and body-based information when studying distance perception.

## Ethics Statement

All procedures performed in this study involving human participants were in accordance with the 1964 Helsinki Declaration and its later amendments or comparable ethical standards, and were reviewed and approved by the Scientific and Ethics Committee of Epsylon Laboratory EA4556, University Paul Valéry of Montpellier.

## Author Contributions

DB, LB, and RJ brought the main idea of the research. RJ, TC, and VM conducted the experiments, collected the data, made the statistical analyses, and wrote the manuscript. NM and RP-G contributed during the writing phase.

## Conflict of Interest Statement

The authors declare that the research was conducted in the absence of any commercial or financial relationships that could be construed as a potential conflict of interest.

## Supplementary Material

TABLE S1 Raw data of Experiment 1 and Experiment 2.

TABLE S2 Bias ratios of Experiment 1 and Experiment 2.
